# P-584. Tailored telehealth behavioral health coaching demonstrates feasibility and potential in overcoming barriers to HIV treatment outcomes

**DOI:** 10.1093/ofid/ofae631.782

**Published:** 2025-01-29

**Authors:** Titilola Labisi, Renae Furl, Deanna M Hansen, Lance L Burwell, Rachelle Carr, Nada Fadul

**Affiliations:** Kaiser Permanente, Los Angeles, California; University of Nebraska Medical Center, Omaha, Nebraska; University of Nebraska Medical Center, Omaha, Nebraska; University of Nebraska Medical Center, Omaha, Nebraska; University of Nebraska Medical Center, Omaha, Nebraska; University of Nebraska Medical Center, Omaha, Nebraska

## Abstract

**Background:**

People with HIV (PWH) face challenges, including behavioral health, stigma, financial constraints, homelessness and homophobia that negatively impact their HIV care. Unfortunately, limited, tailored programs exist to address these problems. Additionally, transportation and time constraints may hinder participation in available programs. Thus, Nebraska’s Specialty Care Center implemented the Intervention to Telehealth and Text to Improve Engagement in Care (i2TEC), a Health Resources and Services Administration (HRSA) HIV/AIDS Bureau-funded evidence-informed intervention aimed to address the intersection of mental health, substance use, and HIV. Here, we describe i2TEC program and its early impact on viral suppression (VS) among PWH in Nebraska.

**Methods:**

Delivered by a licensed behavioral health therapist (BHT), participants receive 12 weekly behavioral health virtual coaching sessions. Participants could choose from various topics such as HIV care, mental health, relationships, sexuality, housing, and lifestyle. Then, they collaboratively work with the BHT to identify barriers to their HIV care and establish goals to overcome them. Participation in i2TEC requires being an HIV diagnosed adult not fully engaged in HIV care.

**Results:**

Of the 50 participants enrolled in i2TEC, 84% were males, and 50% were non-Hispanic Whites. At baseline, 64% had a positive screen for substance use, 54% had a positive screen for depression or anxiety, and 71% did not have durable VS. Sixty percent had follow-up viral load labs, of which 97% had achieved VS within one-year post-enrollment. Participants' most identified discussion topics were lifestyle/health and socioeconomic needs (housing, food, and transportation). Overall, 95% would recommend the program, 86% agreed that i2TEC helped improve their mental health, and 76% reported that the program improved their medication adherence.

**Conclusion:**

Preliminary findings from this tailored telehealth behavioral health program demonstrate feasibility and potential for improving HIV outcomes and overcoming individual barriers to VS. Through the delivery of i2TEC, we identified priority coaching needs among PWH. Future studies focusing on the topics identified by participants and the long-term impact of such programs are recommended.

**Disclosures:**

**Nada Fadul, MD**, ViiV Healthcare: Advisor/Consultant|ViiV Healthcare: Grant/Research Support

